# PINK1 deficiency in β-cells increases basal insulin secretion and improves glucose tolerance in mice

**DOI:** 10.1098/rsob.140051

**Published:** 2014-05-07

**Authors:** Emma Deas, Kaisa Piipari, Asif Machhada, Abi Li, Ana Gutierrez-del-Arroyo, Dominic J. Withers, Nicholas W. Wood, Andrey Y. Abramov

**Affiliations:** 1 Department of Molecular Neuroscience, UCL Institute of Neurology, Queen Square, London WC1N 3BG, UK; 2 Metabolic Signalling Group, Medical Research Council (MRC), Clinical Sciences Centre, Imperial College London, London W12 0NN, UK; 3 Department of Neuroscience, Physiology and Pharmacology, UCL, Gower Street, London WC1E 6BT, UK; 4 UCL Clinical Physiology, Wolfson Institute for Biomedical Research, Division of Medicine, London WC1E 6BT, UK

**Keywords:** PINK1, Parkinson's disease, β-cells, insulin, diabetes

## Abstract

The Parkinson's disease (PD) gene, *PARK6*, encodes the PTEN-induced putative kinase 1 (PINK1) mitochondrial kinase, which provides protection against oxidative stress-induced apoptosis. Given the link between glucose metabolism, mitochondrial function and insulin secretion in β-cells, and the reported association of PD with type 2 diabetes, we investigated the response of PINK1-deficient β-cells to glucose stimuli to determine whether loss of PINK1 affected their function. We find that loss of PINK1 significantly impairs the ability of mouse pancreatic β-cells (MIN6 cells) and primary intact islets to take up glucose. This was accompanied by higher basal levels of intracellular calcium leading to increased basal levels of insulin secretion under low glucose conditions. Finally, we investigated the effect of PINK1 deficiency *in vivo* and find that PINK1 knockout mice have improved glucose tolerance. For the first time, these combined results demonstrate that loss of PINK1 function appears to disrupt glucose-sensing leading to enhanced insulin release, which is uncoupled from glucose uptake, and suggest a key role for PINK1 in β-cell function.

## 2. Introduction

Type 2 diabetes is a common metabolic disorder estimated to affect over 256 million adults worldwide [1]. The predominant mechanism underlying this disease is insulin resistance in peripheral metabolic tissues such as muscle, liver and adipose tissue accompanied by a relative deficit in insulin secretion [2,3]. Under normal circumstances, the alpha (α) and beta (β) islet cells in the pancreas monitor blood glucose levels. When an increase in blood glucose is detected, these cells take up glucose via the GLUT2 glucose transporter leading to an increase in glucose metabolism within the cell [4]. The subsequent increase in mitochondrial substrate availability drives the electron transport chain, increases intracellular ATP levels and induces closure of the ATP-sensitive potassium channel (K^+^-ATP) at the plasma membrane. When the K^+^-ATP channel closes, it induces depolarization of the plasma membrane (*V*_m_), which activates the voltage-dependent calcium channels and induces a calcium influx into the cell. Increased cytosolic calcium ([Ca^2+^]_c_) concentrations then stimulate the secretion of insulin which, in turn, activates glucose transporters in insulin-sensitive cells to allow cells and tissues throughout the body to take up glucose as an energy source [5].

PTEN-induced putative kinase 1 (PINK1) is a mitochondrially targeted serine/threonine kinase which protects cells against stress-induced apoptosis [6]. Homozygous mutations in the PINK1 gene (*PARK6*) and loss of PINK1 function have been shown to be an underlying cause of early onset autosomal recessive Parkinson's disease (PD) [7]. In previous studies, we and others have demonstrated that loss of or impaired PINK1 function in neurons results in a series of mitochondrial abnormalities. These include impaired calcium buffering capacity due to inhibition of calcium efflux in the mitochondria, increased reactive oxygen species (ROS) production through NADPH oxidase, reduced mitochondrial membrane potential, inhibition of the sodium calcium (Na^+^/Ca^2+^) exchanger and impaired glucose transport into the cell [8,9]. However, we have also shown that different cell types, i.e. muscle cells, which are highly dependent on mitochondria, can compensate for loss of PINK1 through increasing glycolysis which overcomes the ATP deficit and improves cellular calcium buffering capacity [10].

Interestingly, reduced transcription of the *PARK6* gene, encoding PINK1, has been reported in type 2 diabetic (T2DM) patients implicating PINK1 in glucose metabolism [11]. In addition, while the PINK1 N521T variant has been directly linked to an increased risk of developing T2DM in a northern Chinese population [12], subsequent genome-wide association studies have thus far not supported a role for PINK1 as a risk factor for T2DM [13]. However, the consequences of PINK1 deficiency on islet cell function and hence the mechanism by which PINK1 dysfunction could contribute to T2DM have yet to be investigated. We therefore used a combination of PINK1 siRNA, primary PINK1 knockout (KO) intact islets and PINK1 KO mice to determine the effect of PINK1 deficiency on β-cell function.

## 3. Results

### 3.1. PINK1 is expressed in MIN6 cells and isolated wild-type islets

**Figure 1.**
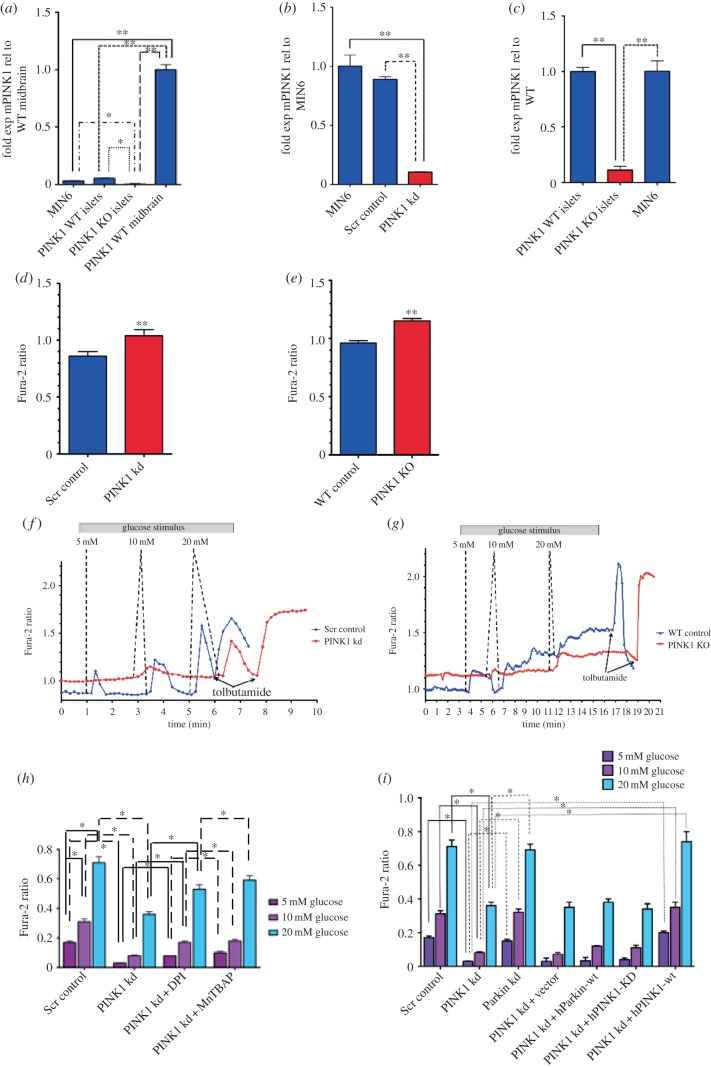
Assessment of cytosolic calcium levels in PINK1-deficient β-cells. (*a*) PINK1 expression in MIN6 cells and isolated PINK1 WT and KO islets compared with WT midbrain. *p < 0.05 and **p < 0.005, Mann–Whitney *U*-test. (*b*) RT-PCR confirmation of PINK1 knockdown levels obtained via siRNA treatment. *p* < 0.005, paired *t*-test. (*c*) RT-PCR confirmation of PINK1 levels in PINK1 WT and KO islets. *p* < 0.005, Mann–Whitney *U*-test. (*d*) Basal levels of Fura-2 ratio in Scr control versus PINK1 kd siRNA-treated cells. *p* < 0.005, paired *t*-test. (*e*) Basal levels of Fura-2 ratio in WT control versus PINK1 KO islets, *p* < 0.005, Mann–Whitney *U*-test. (*f*) Representative traces showing measurement of Fura-2 ratio alteration induced by glucose stimuli in Scr control versus PINK1 kd cells. (*g*) Representative traces measuring alteration of Fura-2 ratio in WT control versus PINK1 KO islets, respectively. *p* < 0.005, paired *t*-test. (*h*) Alteration of Fura-2 ratio in Scr control and PINK1 kd cells with increasing glucose stimuli and improvement of PINK1 kd cell response with DPI or MnTBAP treatment. *p* < 0.005, paired *t*-test. (*i*) Alteration of Fura-2 ratio in Scr control and PINK1 kd cells, but not Parkin kd, with increasing glucose stimuli and rescue of PINK1 kd cell response with PINK1-wt re-expression. *p* < 0.05, paired *t*-test.

To assess whether loss of PINK1 function would potentially have an effect on islet cell function, we sought to determine whether PINK1 was expressed in islet cells. Owing to continuing problems assessing mouse PINK1 by western blot, we assayed MIN6 cells and isolated PINK1 wild-type (WT) and KO islets for PINK1 expression by RT-PCR. PINK1 WT mouse midbrain (known to express high levels of PINK1) was used as a control. Figure 1*a* demonstrates that PINK1 transcripts are present in both MIN6 cells and isolated WT islets, but these levels are significantly reduced by comparison with transcript levels in the midbrain (*n* = 4, *p < 0.01 and **p < 0.0006).

### 3.2. PINK1-deficient β-cells display impaired calcium homeostasis and elevated cytosolic calcium

Given the close link between intracellular calcium levels and the stimulation of insulin secretion in pancreatic β-cells, we assessed the basal levels of [Ca^2+^]_c_ in PINK1-deficient β-cells. Two cellular systems were assessed: knockdown (kd) mouse pancreatic β-cells (MIN6 cells) which had been treated with mouse PINK1 (mPINK1) or scramble (Scr) control siRNA, and intact islets isolated from PINK1 WT and KO littermates (aged six months). RT-PCR analysis of PINK1 levels in both KO islets and in MIN6 cells treated with mPINK1 siRNA showed a significant decrease in detectable levels of mPINK1 in both systems (figure 1*b*,*c*, *n* = 4, *p* < 0.003). [Ca^2+^]_c_ was measured using the Fura-2 indicator. Upon initial examination, we noted that the basal intracellular [Ca^2+^]_c_ concentration in PINK1-deficient cells was significantly higher compared with controls (1.04 ± 0.05 Fura-2 ratio (*n* = 86 cells) in PINK1 kd compared with 0.86 ± 0.04 Fura-2 ratio in control (*n* = 91); *p* < 0.001; figure 1*d*). In agreement, the PINK1 KO intact islets also had a significantly higher basal level of [Ca^2+^]_c_ compared with WT controls (1.15 ± 0.02 Fura-2 ratio (*n* = 9 islets) in PINK1 KO islets compared with 0.96 ± 0.02 in WT control (*n* = 10); *p* < 0.001; figure 1*e*). We subsequently examined the effects on [Ca^2+^]_c_ levels after glucose stimulation. Application of 5, 10 and 20 mM glucose to Scr control MIN6 cells and WT islets induced a dose-dependent ‘peak-like’ increase in [Ca^2+^]_c_ as expected (*n* = 5 experiments; figure 1*f* and *g*, respectively). By contrast, PINK1 kd MIN6 cells or KO islets showed no increase in [Ca^2+^]_c_ when exposed to 5 mM glucose and demonstrated a significantly smaller [Ca^2+^]_c_ increase in response to both 10 (*p* < 0.01) and 20 mM (*p* < 0.001) glucose (*n* = 4 experiments; figure 1*f* and *g*, respectively). Notably, the difference between basal calcium levels in PINK1 kd cells and KO islets was comparable to the calcium signal induced in the control cells and WT islets in response to the 5 mM glucose stimulus (figure 1*f*,*g*). As previously mentioned, calcium influx in β-cells, in response to glucose stimuli, is typically regulated by K^+^-ATP channels. To determine whether the normal calcium signalling pathway through the K^+^-ATP channels was impaired by PINK1 deficiency, MIN6 cells and islets were treated with 100 µM tolbutamide (which inhibits activation of K^+^-ATP channels and should induce a classical high [Ca^2+^]_c_ influx). Application of tolbutamide induced comparable calcium signals between PINK1-deficient and WT controls in both MIN6 cells and intact islets (figure 1*f* and *g*, respectively). This suggests that, despite the alteration in glucose-stimulated calcium influx, PINK1-deficient β-cells have a normal calcium signal through the K^+^-ATP-dependent pathway.

We have previously shown that PINK1 deficiency in neurons induced high levels of cellular ROS through NADPH oxidase [8,14]. To ascertain if the increased basal [Ca^2+^]_c_ in PINK1-deficient β-cells was also induced by elevated levels of cellular ROS through NADPH oxidase activity, the ability of PINK1 kd β-cells to respond to glucose stimuli was reassessed after treatment with 0.5 μM of the NADPH inhibitor diphenylene iodonium (DPI, *n* = 3 experiments), and the anti-oxidant MnTBAP (a ROS scavenger, *n* = 3 experiments). After treatment, PINK1 kd cells showed a significant improvement in their ability to respond to 5, 10 and 20 mM glucose, but this increase was not sufficient to restore the levels back to those observed in control cells (figure 1*h*).

In PD, PINK1 is known to function upstream in the same pathway as another PD-related protein called ‘Parkin’, where overexpression of Parkin can compensate for PINK1 deficiency [15]. To confirm our observations were therefore specific to loss of PINK1, we re-examined the effects on intracellular calcium after knockdown of mouse Parkin via siRNA and also performed rescue experiments in the PINK1 kd cells by transfecting in either vector control, human PINK1-wild-type (hPINK1-wt), human PINK1-kinase dead (hPINK1-KD) or human Parkin (hParkin-wt). Figure 1*i* shows that only expression of hPINK1-wt was able to rescue the effects of the aberrant calcium signal in PINK1 kd cells, suggesting that these effects are a direct consequence of PINK1 deficiency and are dependent on PINK1 kinase activity (*n* = 3, *p* < 0.05). Confirmation that PINK1 kd had no effect on endogenous Parkin levels and the efficiency of siRNA-mediated Parkin knockdown are shown in the electronic supplementary material, figure S1.

### 3.3. Loss of PINK1 results in mitochondrial dysfunction in β-cells

**Figure 2.**
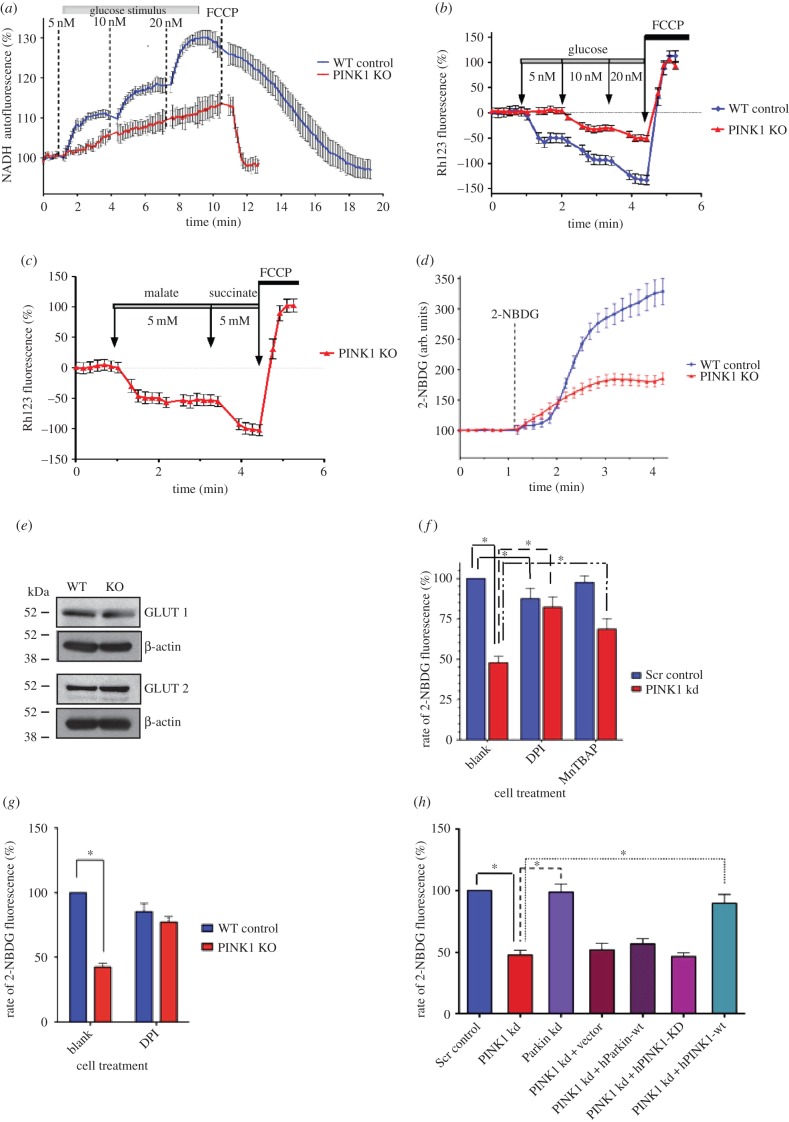
PINK1 deficiency impairs mitochondrial function and glucose-sensing. (*a–d*) Representative traces measuring alteration of NADH autofluorescence, rhodamine 123 fluorescence and 2-NBDG uptake in WT control versus PINK1 KO islets, respectively. *p* < 0.005, Mann–Whitney *U*-test. (*e*) GLUT1 and GLUT2 transporter expression in WT and PINK1 KO islets. (*f*) Alteration of 2-NBDG uptake rate in Scr control versus PINK1 kd cells at basal levels and after DPI or MnTBAP treatment. *p* < 0.001, *p* < 0.00001 and *p* < 0.005, respectively, paired *t*-test. (*g*) Alteration of 2-NBDG uptake rate in WT control versus PINK1 KO intact islets at basal levels and after DPI treatment. *p* < 0.005 and *p* < 0.001, respectively; Mann–Whitney *U*-test. (*h*) Alteration of 2-NBDG uptake rate in PINK1 kd versus Scr control and Parkin kd cells at basal levels and rescue of PINK1 kd uptake rate through re-expression of PINK1-wt. *p* < 0.005, paired *t*-test.

Under normal circumstances, application of glucose to cells induces an increase in glucose metabolism, which, in turn, increases the concentration of available mitochondrial substrates [16]. The substrates are used by mitochondria, and this increases a number of mitochondrial characteristics such as the production of NADH and an increase in mitochondrial membrane potential (ΔΨm) [17]. As PINK1 is known to be essential for maintaining normal mitochondrial function in neurons, we assessed mitochondrial function in the islets. In our experiments, application of increasing concentrations of glucose induced a dose-dependent increase of NADH autofluorescence in WT islets (*n* = 12; figure 2*a*). However, the application of the same glucose solutions induced a significantly smaller response in PINK1-deficient islets (*n* = 10; figure 2*a*). A lower level of mitochondrial substrates, such as NADH, in PINK1-deficient cells should make them less sensitive to changes in ΔΨm. To confirm this, we assessed alterations in ΔΨm in response to glucose stimuli using rhodamine 123 (Rh123) [18]. Figure 2*b* shows that application of 5, 10 and 20 mM of glucose induces a step-like increase in ΔΨm in WT islets which registers as a decrease in Rh123 fluorescence. Application of the same glucose solutions to PINK1 KO islets induced a significantly smaller hyperpolarization in ΔΨm. As a control, complete depolarization of the cells was induced with 1 µM FCCP and generated a maximal increase in Rh123 fluorescence. Importantly, application of mitochondrial substrates such as malate or methyl succinate induced a profound mitochondrial hyperpolarization in both cell types (figure 2*c*), suggesting that the mitochondria in PINK1-deficient islets are not damaged and have a fully functional response when mitochondrial substrates are available. Considering this, our results suggest that PINK1-deficient β-cells have impaired glucose delivery or glycolysis.

### 3.4. PINK1 deficiency impairs glucose uptake in β-cells

To determine whether glucose uptake was impaired in PINK1-deficient β-cells, we used the fluorescent glucose analogue 2-NBDG (which is used to monitor glucose uptake in live cells) and repeated the experiment described above. Our results show that at basal levels, the ability of PINK1 kd β-cells and intact KO islets to take up glucose is significantly impaired compared with Scr control cells or WT islets (47.8 ± 3.9% of control, *n* = 4 experiments; *p* < 0.001; figure 2*f* and 42.4 ± 3.1% of control islets; *n* = 9; *p* < 0.001; figure 2*d,* respectively). To ensure this alteration was not due to altered expression of either the GLUT1 or GLUT2 transporters, the expression of both transporters was assessed by western blot and found to be comparable between both WT and PINK1 KO islets (figure 2*e*). Upon treatment with DPI, PINK1-deficient cells and islets show a significant improvement in their ability to take up glucose, suggesting that increased ROS levels, through NADPH oxidase activity, may underlie the abnormal [Ca^2+^]_c_ (from 47.8 ± 3.9% to 82.4 ± 6.4%, *n* = 69; *p* < 0.00001; figure 2*f* and from 42.4 ± 3.1% to 77.2 ± 4.1%, *n* = 9 islets; *p* < 0.001; figure 2*g*). Treatment of PINK1 kd cells with MnTBAP also significantly improved glucose uptake (from 47.8 ± 3.9% to 68.7 ± 6.3%, *n* = 37; *p* < 0.005; figure 2*f*). However, while DPI treatment was able to rescue PINK1 kd glucose uptake back to levels comparable to those observed in Scr cells, it could not rescue the alteration in [Ca^2+^]_c_ to the same extent (compare figure 2*f* with figure 1*h*). To confirm that inhibition of glucose uptake was specifically due to loss of PINK1, we repeated the 2NDBG experiments in MIN6 cells after siRNA-induced mouse Parkin kd and via rescue experiments in the PINK1 kd β-cells. The rescue experiments were done by transfecting in either vector control, hPINK1-wt, hPINK1-KD or hParkin-wt. Loss of Parkin was found to have no effect on glucose uptake compared with vector or Scr control cells, and only expression of hPINK1-wt was able to restore glucose uptake in PINK1-deficient β-cells (figure 2*h, n* = 42; *p* < 0.005).

Interestingly, incubation of Scr control cells with 0.5 μM of DPI induced the opposite effect and demonstrated a slight inhibition of glucose uptake (figure 2*f*), suggesting that ROS can be one of the regulators of this transporter.

### 3.5. Loss of PINK1 increases basal levels of insulin secretion

**Figure 3.**
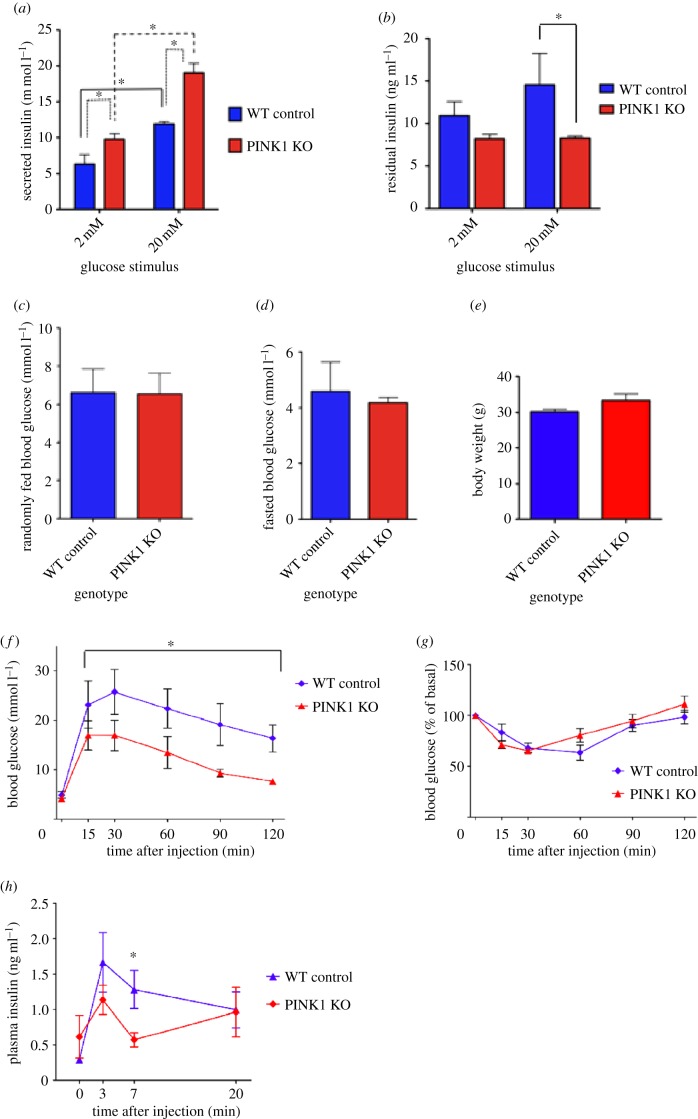
PINK1 KO mice suffer from glucose tolerance and altered insulin secretion *in vivo*. (*a*) Concentration of insulin secreted by WT and KO PINK1 isolated islets under low and high glucose stimuli. *p* < 0.005, Mann–Whitney *U*-test. (*b*) Residual concentration of insulin extracted from WT and KO islets. *p* < 0.05, Mann–Whitney *U*-test. (*c,d*) Basal blood glucose concentrations in PINK1 WT and KO mice in response to random feeding or overnight starvation, respectively. (*e*) Comparison of body weight between PINK1 WT and KO animals. (*f*) Response of PINK1 WT and KO animals to a GTT. Two-way ANOVA with Bonferroni multiple comparison test with repeated measures. *p* > 0.005, Mann–Whitney *U*-test. (*g*) Response of PINK1 WT and KO animals to an ITT. (*h*) Assessment of blood insulin levels in PINK1 WT and KO animals in response to a GSIS test. *p* > 0.005, Mann–Whitney *U*-test.

To determine the impact of PINK1 deficiency on insulin release, we measured the level of insulin secreted by WT control and PINK1 KO intact islets under low and high glucose conditions. As expected, the WT islets secreted significantly more insulin in response to the high 20 mM glucose stimulus than the low 2 mM stimulus (*n* = 6; *p* < 0.05; figure 3*a*). By contrast, PINK1 KO islets secreted significantly higher levels of insulin than their WT counterparts in response to 2 mM glucose and, notably, the level secreted was comparable to the WT islet response to 20 mM glucose (*n* = 6; *p* < 0.05; figure 3*a*). When exposed to high glucose, the PINK1 KO islets also secreted significantly more insulin compared with those observed under low glucose conditions (*n* = 6; *p* < 0.005; figure 3*a*). This confirms our previous observations in MIN6 cells and intact islets demonstrating that PINK1-deficient cells can still respond to high concentrations of glucose (figure 1*f*,*g*). To determine whether the difference in insulin secretion between WT and PINK1 KO islets was due to altered total levels of insulin, the residual insulin was extracted from the islets by acid ethanol and measured. Notably, the total concentration of insulin (secreted plus extracted) was comparable between WT and PINK1 KO islets (figure 3*b*, *n* = 6; *p* < 0.05).

**Figure 4.**
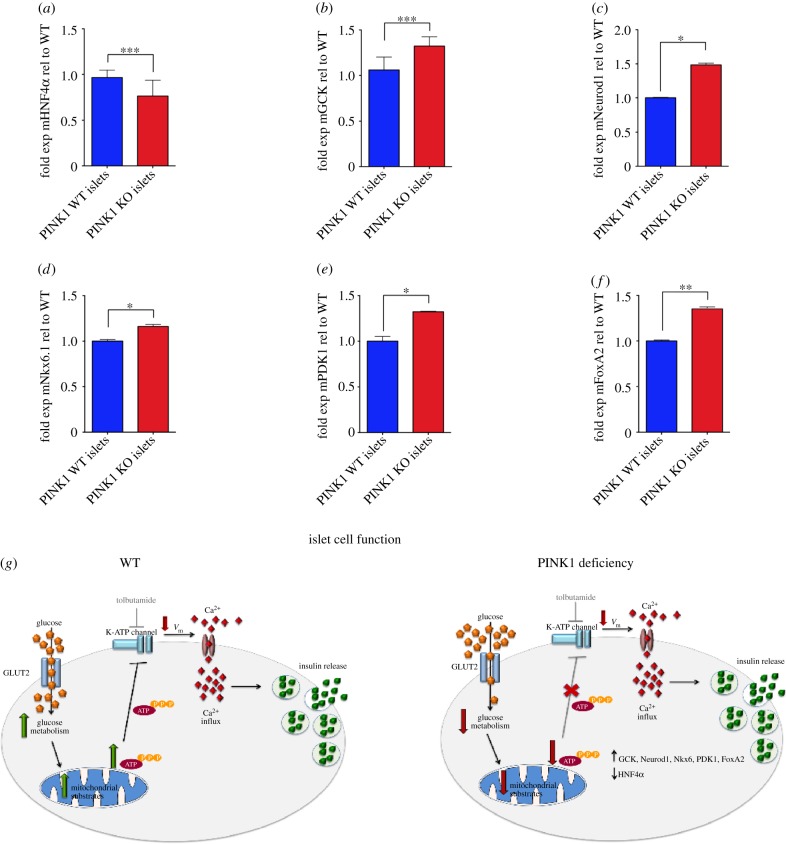
PINK1 mice display altered glucose homeostasis gene transcription in intact islets. (*a–f*) RT-PCR assessment of gene transcription in PINK1 KO versus WT islets showing a downregulation of HNF4α and an upregulation of GCK, Neurod1, Nkx6.1, PDK1 and FoxA2, respectively. *p* < 0.05, Mann–Whitney *U*-test. (*g*) Illustration of how PINK1 deficiency results in altered insulin secretion and gene regulation in intact islets.

As the final stage of our investigation, we used six month old male PINK1 WT and KO mice to assess the effect of PINK1 deficiency on islet function *in vivo*. Blood glucose levels of both PINK1 WT and KO mice showed no discernable differences after random feeding or 16 h starvation (figure 3*c,d*). In addition, body weight was comparable (figure 3*e*) and preliminary observations indicated no difference in food or water consumption (data not shown). A glucose tolerance test (GTT) revealed that while PINK1 WT and KO mice initially show a comparable increase in blood glucose levels, the levels in PINK1 KO mice peak at a lower level and decrease significantly faster than the WT littermates (figure 3*f*, *p* < 0.05). However, the response to an insulin tolerance test (ITT) was comparable and showed an initial drop in blood glucose levels followed by recovery (figure 3*g*). A glucose-stimulated insulin secretion (GSIS) experiment revealed that, as in our islet experiments, PINK1 KO mice have a higher basal level of insulin in the bloodstream (figure 3*h*). This, however, did not reach significant levels compared with WT. Upon injection of glucose, the PINK1 KO mice were initially stimulated to secrete insulin into the blood, but after 3 min, the level secreted was lower than that observed in WT animals (figure 3*f*). At 7 min post-injection, the blood insulin levels in the PINK1 KO mice had returned to baseline, whereas the WT blood insulin levels, although reduced, remained significantly higher than the basal reading (*p* < 0.05). However, insulin secretion in the KO animals increased to levels comparable to WT littermates 20 min post-injection (figure 3*f*). These combined results suggest that PINK1 deficiency results in improved glucose tolerance *in vivo* and also indicate that, *in vivo*, there appears to be a trend towards increased basal blood insulin levels with a reduced initial secretion response. In addition to these studies, RT-PCR analysis of several key genes involved in glucose homeostasis (GSK3β, Pecam1, HNF1α, HNF4α, ECAD, GCK, Neurod1, Nkx6.1, FoxA2, PDK1, Pfkp, ALDOB, GAPDH and AKT) revealed that while HNF4α was downregulated in PINK1 KO islets, GCK, Neurod1, Nkx6.1, PDK1 and FoxA2 were all upregulated compared with WT age-matched controls (figure 4*a*–*e* and *f*, respectively, *n* = 4; for HNF4α and GCK *p* < 0.0006, for FoxA2 and PDK1 *p* < 0.004, for Nkx6.1 and Neurod1 *p* < 0.02). The remaining genes examined showed no significant alteration between genotypes (electronic supplementary material, figure S2).

## 4. Discussion

Mutations within the PINK1 gene have been extensively shown to be responsible for early-onset autosomal recessive PD [7,19]. PINK1 is a mitochondrial protein, and there is now overwhelming evidence that mitochondrial dysfunction is a key player in PD [20,21]. However, while the death of dopaminergic neurons in the substantia nigra of the brain is the most prominent disease presentation in patients with PINK1 mutations, recent reports have implicated PINK1 gene dysfunction or dysregulation as a potential risk factor for the metabolic disorder T2DM [11,12]. The aim of this study was to assess the functional significance of PINK1 deficiency in β-cells and islets. Our results show that in the pancreas, normal β-cell function is impaired through PINK1 deficiency leading to higher basal levels of insulin secretion *in vitro* and a trend towards increased basal plasma insulin *in vivo*. Using MIN6 cells and intact islets from PINK1 KO mice, we have demonstrated that this increase is independent of glucose levels and is instead induced by higher basal calcium levels in the cells. We have additionally shown that glucose uptake is significantly inhibited in PINK1 kd β-cells and KO islets compared with WT controls. This leads to impaired mitochondrial function and calcium signalling in response to low glucose concentrations. Through bypassing the glucose metabolism pathway and simply supplying the islets with alternative mitochondrial substrates, for example, malate and methyl succinate, we have shown that the normal β-cell pathway to insulin secretion, including regulation of calcium influx through the K^+^-ATP channel, is not impaired by a PINK1 deficit. Together, these data indicate that the glucose transporter is inhibited, but not fully impaired, in these cells. In support of this, our combined data show that high concentrations of glucose can still stimulate additional insulin release from PINK1-deficient cells through the glucose stimulation pathway. Loss of PINK1 function therefore appears to uncouple GSIS during low levels of glucose stimuli. In addition, through rescue experiments using inhibitors or anti-oxidants, we have shown that inhibition of the glucose transporter is due to ROS production by NADPH oxidase. One intriguing result obtained was the mild inhibition of the glucose transporter in WT β-cells with the NADPH oxidase inhibitor, suggesting that this enzyme is essential for normal maximal function of the glucose transporter in these cells.

Our *in vivo* results demonstrate that the PINK1 KO animals have improved glucose tolerance. One explanation for these findings is that the increased basal insulin level is sufficient to stimulate the presentation of glucose transporters at the cell surface [22] and therefore primes the peripheral tissues to take up glucose from the blood at a faster rate. While our *in vitro* data would support this hypothesis, the insulin increase did not reach significance in our *in vivo* study when compared with WT controls. This could be owing to compensation mechanisms keeping insulin levels below detrimental physiological levels or owing to experimental procedure. We did, however, observe a significant upregulation of both GCK and PDK1 transcription (known to be regulated by insulin), suggesting that the levels of insulin *in vivo* may still be sufficient to elicit an abnormal physiological effect. Interestingly, we also observed an upregulation of Neurod1 and NKx6.1, which are known to increase transcription of the insulin gene [23,24] and a reduction in HNF4α transcription, known to occur during hyperinsulinemia (excess levels of insulin in the blood). However, these alterations could be due to compensatory/homeostatic mechanisms attempting to regulate insulin levels in the blood. The upregulation of FoxA2 could also account for the observed reduction of HNF4α transcription, as these are reportedly linked [25]. Finally, we cannot exclude additional factors such as a reduction in hepatic gluconeogenesis that could result in the *in vivo* improved glucose tolerance. While we have yet to identify the underlying *in vivo* mechanism behind the increased blood glucose clearance, the results of the ITT indicate that the peripheral insulin sensitivity functions normally.

In the broader setting of T2DM, the predominant cause of T2DM is frequently insulin resistance. It is therefore plausible that the reduced transcription of PINK1, reported in T2DM patients by Scheele *et al.* [11], could be a compensatory mechanism attempting to increase basal insulin secretion, so the peripheral tissues respond to a glucose stimulus. Current T2DM therapeutics, specifically sulfonylureas, work by this mechanism. However, we cannot exclude the possibility that the reduction in PINK1 levels, through inducing increased levels of basal insulin secretion, could contribute to the development of insulin resistance over time. In order to determine whether the reduced PINK1 levels reported by Scheele and colleagues are a cause or effect of T2DM, studies using inducible β-cell-specific PINK1 KO studies in mice is required. Furthermore, it would be of interest to determine the effect of the N521T variation on PINK1 transcription levels, protein levels and function to see if this mutation results in reduced levels of functional PINK1.

In the context of PD, a study reported that 50–80% of PD patients exhibit abnormal glucose tolerance [26]. Notably, while this study was conducted nearly two decades ago, there is now increasing evidence that T2DM and PD may commonly co-occur [11,12], and a retrospective study of T2DM patients reports that diabetes is associated with a significantly higher risk of developing PD [27]. Given the link between the regulation of insulin and dopamine in the body, this is perhaps not surprising. Specifically, insulin resistance has been reported to reduce both nigrostriatal dopamine release and dopamine clearance in the brain [28], whereas insufficient insulin secretion results in reduced dopamine transporter and tyrosine hydroxylase mRNA in the substantia nigra [29]. Insulin has also been reported to have multiple neuroprotective functions in the brain [30], and more recently, increased insulin levels have been shown to directly affect the firing rate of mouse midbrain tyrosine hydroxylase positive neurons—the loss of which results in PD in humans [31]. These combined effects of insulin dysregulation on the dopaminergic neuronal system may partly explain why diabetes is a risk factor for PD, but our finding that a deficiency of the PD-associated protein PINK1 can directly alter insulin secretion from islets suggests that these two diseases could be linked mechanistically.

The interconnected signalling effects of both insulin and dopamine therefore need to be addressed in the context of patient treatment and management. When compared with patients who suffered from PD alone, PD patients with diabetes were found to have accelerated progression of both motor and cognitive neurodegenerative symptoms [32,33]. Interestingly, bromocriptine (a potent dopamine receptor agonist) has been shown to improve insulin sensitivity in rodent studies [34], whereas exposure of animals to levodopa (a common PD therapeutic) induces an increase in blood glucose levels [35]. More recently, the diabetic drug Actos (pioglitazone) was highlighted as a potential PD therapeutic, because the drug activates the PGC-1α protein pathway, and genes regulated by PGC-1α are known to be present at abnormally low levels in PD brain [36]. It will therefore be of significant interest to the PD field to assess additional established diabetic therapeutics as novel PD treatments and whether aggressive treatment of T2DM can reduce the incidence of PD in general.

## 5. Material and methods

### 5.1. Animals

PINK1-deficient mice, originally generated by Lexicon Genetics Inc. (The Woodlands, TX), were obtained under MTA through Julian Downward, CRUK, London. Mice were maintained on a 12 L : 12 D cycle with free access to water and food. Animal husbandry and experimental procedures were performed in full compliance with the United Kingdom Animal (Scientific Procedures) Act of 1986 and with approval of the University College London Animal Ethics Committee. Animals were genotyped by PCR amplification of ear biopsy material in the following manner: genomic DNA was isolated from biopsy tissue using the extract-N-Amp tissue PCR kit (Sigma) and amplified with primers PINK1–26 (5′-CTGCCCTCAGGGTCTCTAATGC-3′), PINK1–27 (5″-GGAAGGAGGCCATGGAAATTGT-3′) and Neo (5′-GCAGCGCATCGCCTTCTATC-3′). PCR conditions were 94°C denaturation—45 s, 60°C annealing—45 s and 72°C extension—45 s. This cycle was repeated 30 times.

### 5.2. MIN6 cell culture and siRNA transfections

Murine MIN6 β-cells were routinely cultured at 37°C with 5% CO_2_ in Dulbecco's modified Eagle medium supplemented with 10% fetal bovine serum (FBS) Gold (PAA Laboratories). For imaging experiments, MIN6 β-cells were plated at a density of 300 000 onto poly-l-lysine-coated coverslips and left overnight to adhere. Cells were then transfected with either mouse PINK1 siRNA or scrambled control siRNA (siGENOME pools, Dharmacon) as per the manufacturer's instructions and incubated for 48 h. One hour prior to imaging, cells were cultured in HEPES-buffered salt solution (HBSS) supplemented with 20 mmol l^−1^ HEPES and 2 mmol l^−1^ d-glucose.

### 5.3. Islet cell isolation

Mice were killed by cervical dislocation, and islet cells were isolated as previously described [37]. Briefly, the common bile duct was cannulated and its duodenal end occluded by clamping. Liberase solution (2 ml at 0.25 mg ml^−1^ in Krebs–Ringer buffer solution with 100 U ml^−1^ penicillin/100 μg ml^−1^ streptomycin (Pen/Strep)) was injected into the duct to distend the pancreas. The pancreas was then removed, placed in a 15 ml falcon containing 2.5 ml Liberase solution and incubated at 37°C for 12 min. Cold quench buffer (8 ml) was added, and the tube was shaken vigorously to break up the tissue. The dissociated tissue solution was then strained through a 1 mm diameter wire mesh filter into a 50 ml falcon, an additional 10 ml of quench buffer was added and the sample was centrifuged at 1000 r.p.m. for 1 min at 4°C. The supernatant was discarded, and the cell pellet containing the islets was resuspended in 20 ml quench buffer. The centrifugation step was repeated twice. After the final spin, the sides of the falcon tube were dried, 15 ml of cold Ficoll solution (15 ml) was added and the sample was vortexed to resuspend the cells. Cold Ficoll solution was carefully added to the tube followed by 15 ml of cold quench buffer (again added carefully to create a Ficoll gradient). Samples were then centrifuged at 2510 r.p.m. for 22 min at 10°C. The supernatant was carefully removed and passed through a 70 μm filter to retain intact islets. Collected cells were washed three times in 20 ml quench buffer before being rinsed into a 10 cm cell culture dish with RPMI GlutaMAX supplemented with 25 mM HEPES, 10% FBS, Pen/Strep. Islets of similar sizes were either allocated in batches of five into 96-well plates for static insulin secretion experiments or plated onto poly-l-lysine-coated coverslips and left to adhere overnight for imaging studies.

### 5.4. Imaging studies

Fluorescence measurements were obtained on an epifluorescence inverted microscope equipped with a 20×/0.5 fluorite objective. Emitted fluorescence light was reflected through a filter to a cooled CCD camera (Retiga, QImaging, Canada) and digitized to 12-bit resolution. All imaging data were collected and analysed using software from Andor (Belfast, UK).

For measurements of [Ca^2+^]_c_, cells were loaded for 30 min at room temperature with 5 μM of Fura-2 AM in combination with 0.005% pluronic acid in HBSS composed of 156 mM NaCl, 3 mM KCl, 2 mM MgSO_4_, 1.25 mM KH_2_PO_4_, 2 mM CaCl_2_, 10 mM glucose and 10 mM HEPES (pH adjusted to 7.35 with NaOH). DPI was used at a concentration of 0.5 μM and MnTBAP at 200 μM. Traces were normalized to the maximal value of calcium concentration induced upon exposure to ionomycin.

For glucose uptake experiments, the uptake of the fluorescent glucose homologue 2-NBDG (Invitrogen) was measured by the addition of 40 nM 2-NBDG in the presence of 0.25 mM glucose during a period of continuous imaging.

For measurements of ΔΨm, cells were loaded with 2.6 μM (equivalent to 1 μg ml^−1^; Molecular Probes) Rh123 for 15 min at room temperature, and the dye was washed prior to the experiment. Under these loading conditions, Rh123 is non-toxic and gives a reliable and reproducible measure of ΔΨm through the ‘dequench’ of mitochondrial fluorescence, and thus an increase in the Rh123 signal reflects mitochondrial depolarization.

### 5.5. Insulin secretion experiments

Islet cell insulin secretion experiments were conducted as follows. Islets were isolated from animals as described above and cultured in RPMI 1640 GlutaMAX media supplemented with 25 mM HEPES, 10% FCS and Pen/Strep. The following day, islets were transferred to a fresh 35 mm culture dish, and medium was replaced with low glucose secretion buffer (1× KRB solution, 10 mM HEPES, 1 mM CaCl_2_, 0.5% BSA and 2 mM glucose). Islets were incubated at 37°C for 1 h. Five islets of similar size were transferred in 10 μl into 1 well of a 96-well plate. A minimum of six wells per animal subject was plated. The islets were then incubated in 150 μl of low or high glucose secretion buffer (recipe as described above but 20 mM glucose was supplied in the high glucose secretion buffer) for 1 h at 37°C. The 96-well plate was placed on ice and centrifuged at 1000 r.p.m. for 1 min. One hundred millilitres of the supernatant was removed and frozen for assessment. The residual medium was aspirated, and 100 μl of ice-cold acid ethanol was added to the wells and the plate was frozen overnight at −20°C to extract the remaining insulin. Supernatant and acid ethanol samples were assessed for insulin content using the Millipore insulin ELISA.

### 5.6. Glucose tolerance test and glucose-stimulated insulin secretion

Male mice were fasted overnight prior to test initiation. The GTT and GSIS procedures were performed as previously described [38]. Briefly, a tail snip was performed, basal blood glucose levels were measured using glucose test strips (Bauers) and blood was collected to later assess basal insulin levels. Mice were then weighed, injected with 2 g kg^−1^ fresh glucose solution and blood glucose was measured at 3, 7, 15, 20, 30, 60, 90 and 120 min time points post-injection. Additional blood samples to assess insulin levels were collected at 3, 7 and 20 min during the procedure. Blood samples to assess insulin levels were kept on ice before centrifugation at 5130 r.p.m. (300*g*) for 15 min at 4°C. The supernatant was retained and analysed by insulin ELISA.

### 5.7. Insulin tolerance test

Male mice were left for three weeks to recover from the GTT and GSIS procedure prior to the ITT test. The tail snip wound scab was removed, and basal blood glucose was measured as described in the GTT procedure. Male mice were reweighed and injected with a stock solution of human recombinant insulin (0.185 U ml^−1^ in saline). The amount of insulin injected per animal was calculated as follows: body weight (g) × 4 = μl volume of stock insulin solution injected by IP. Blood glucose levels were then assessed at 15, 30, 60, 90 and 120 min post-injection using the glucose test strips as previously mentioned.

### 5.8. Insulin ELISA

Static secreted and blood plasma insulin levels were assessed by ELISA using the rat insulin kit (Millipore) as per the manufacturer's instructions.

### 5.9. RT-PCRs

SYBR Green RT-PCRs were performed using SYBR Select master mix (Invitrogen) and performed on a Corbett Life Sciences Rotor-Gene 6000 platform. Validated mouse RT-PCR primer sequences were obtained via PrimerBank (http://pga.mgh.harvard.edu/cgi-bin/primerbank) and ordered from Invitrogen. PCR conditions were 95°C hold for 5 min, 95°C for 15 s followed by 60°C for 45 s for 45 cycles, then a melt. The specific primer sequences used can be found in the electronic supplementary material, table S1. All data were normalized to two housekeepers: HPRT1 and cyclophilinA.

### 5.10. Statistics

Data are presented as mean ± s.e.m., unless otherwise stated. Statistics were performed using a combination of Origin Pro, GraphPad (PRISM) and Microsoft Excel software. Paired *t*-tests were performed on all MIN6 experiments, the Mann–Whitney *U*-test was performed on all isolated islet studies and two-way ANOVAs with Bonferroni multiple comparison test with repeated measures were performed for *in vivo* studies. Values of *p* < 0.05 are denoted as * whereas ** represents *p* < 0.005, unless stated otherwise.

## Supplementary Material

Supplemental Figure S1: PINK1 and Parkin siRNA knockdown efficiency

## Supplementary Material

Supplemental Figure S2: RT-PCR analysis of glucose homeostasis genes

## Supplementary Material

Supplementary Table 1 - RT-PCR primers
