# Supplementary figures and images for: PINK1 deficiency in β-cells increases basal insulin secretion and improves glucose tolerance in mice
Source: Open Biol. 2014 May 7;4(5):140051. doi: 10.1098/rsob.140051 (PMC4042854; doi:10.1098/rsob.140051)

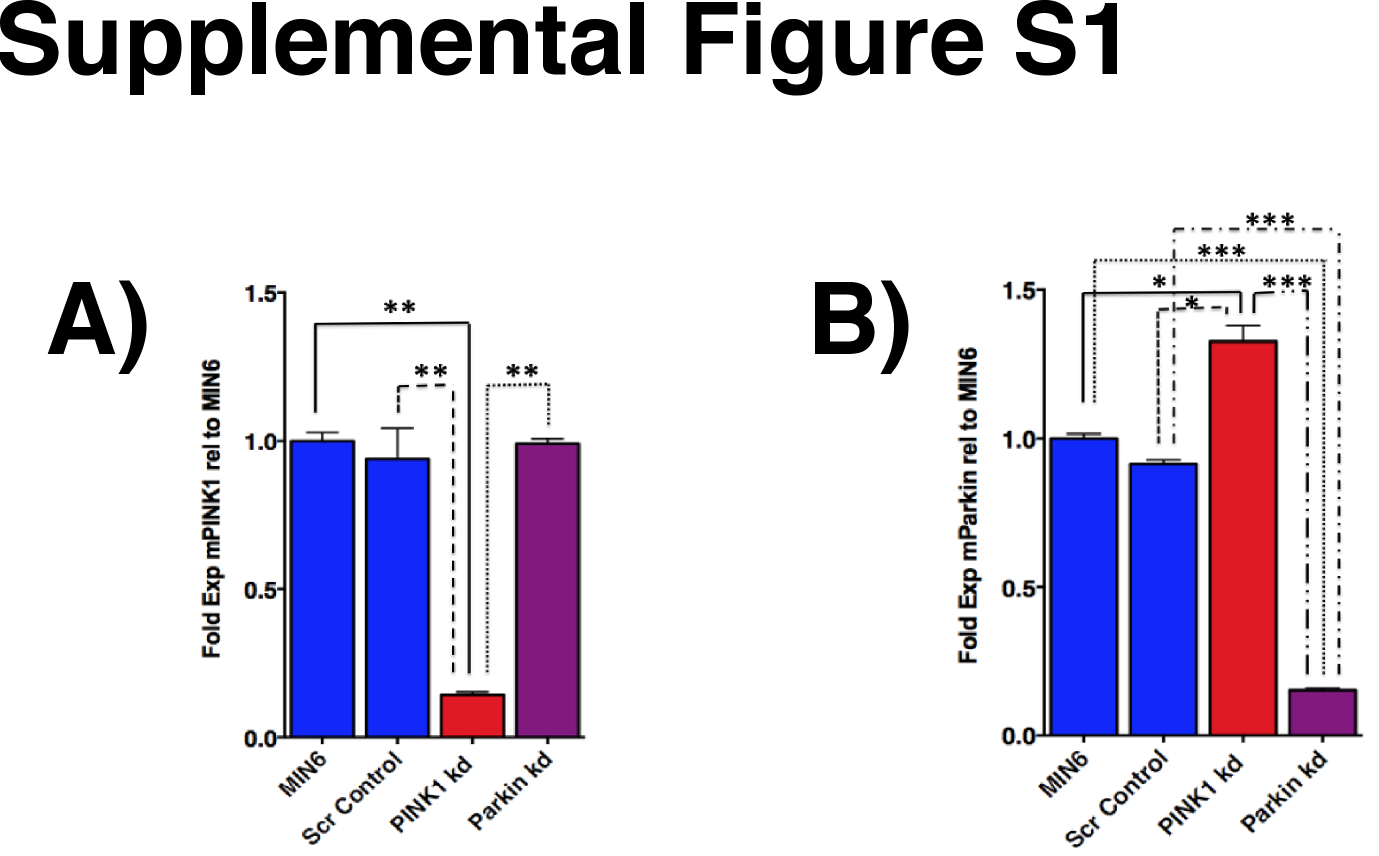

Supplement: Supplemental Figure S1: PINK1 and Parkin siRNA knockdown efficiency [file rsob140051supp1.tif]
