# Supplementary material for: PINK1 deficiency in β-cells increases basal insulin secretion and improves glucose tolerance in mice
Source: Open Biol. 2014 May 7;4(5):140051. doi: 10.1098/rsob.140051 (PMC4042854; doi:10.1098/rsob.140051)
Supplement: Supplementary Table 1 - RT-PCR primers [file rsob140051supp3.docx]

**Supplementary Table 1 - RT-PCR primers**

| **Oligo name** | **Oligo sequence (5' to 3')** | **TM** | **Oligo name** | **Oligo sequence (5' to 3')** | **TM** |
| --- | --- | --- | --- | --- | --- |
| mPINK1fwd2 | GGCTTCCGTCTGGAGGATTAT | 61.1 | mAldobfwd1 | GAAACCGCCTGCAAAGGATAA | 60.9 |
| mPINK1rev2 | AACCTGCCGAGATATTCCACA | 60.9 | mAldobrev1 | GAGGGTCTCGTGGAAAAGGAT | 61.2 |
| mPINK1fwd4 | TTCTTCCGCCAGTCGGTAG | 61.1 | *mAldobfwd2* | *GAAACCGCCTGCAAAGGATAA* | 60.9 |
| mPINK1rev4 | CTGCTTCTCCTCGATCAGCC | 62.1 | *mAldobrev2* | *TCTGAACAGATTTCCCTGGCT* | 60.8 |
| mHprtfwd1 | TCAGTCAACGGGGGACATAAA | 60.8 | mPfkpfwd2 | GGGACCATCATCGGTAGTGC | 62.1 |
| mHprtrev1 | GGGGCTGTACTGCTTAACCAG | 62.4 | mPfkprev2 | GTCCGCTCCACTCCTTTCG | 62.4 |
| mGapdhfwd1 | AGGTCGGTGTGAACGGATTTG | 62.6 | mPfkpfwd4 | GAAACATGAGGCGTTCTGTGT | 60.8 |
| mGapdhrev1 | GGGGTCGTTGATGGCAACA | 62.6 | mPfkprev4 | CCCGGCACATTGTTGGAGA | 62.3 |
| mGapdhfwd4 | AGGTCGGTGTGAACGGATTTG | 62.6 | mPdk1fwd3 | GCACTCCTTATTGTTCGGTGG | 61 |
| mGapdhrev4 | TGTAGACCATGTAGTTGAGGTCA | 60.2 | mPdk1rev3 | CGTCGCAGTTTGGATTTATGCT | 61.5 |
| mParkinfwd2 | CGTGTGATTTTTGCCGGGAAG | 62.4 | mPdk1fwd4 | GGACTTCGGGTCAGTGAATGC | 63 |
| mParkinrev2 | GGTCCACTCGTGTCAAGCTC | 62.5 | mPdk1rev4 | TCCTGAGAAGATTGTCGGGGA | 62.1 |
| mParkinfwd4 | TCTTCCAGTGTAACCACCGTC | 61.4 | mPecam1fwd | ACGCTGGTGCTCTATGCAAG | 62.6 |
| mParkinrev4 | GGCAGGGAGTAGCCAAGTT | 61.3 | mPecam1rev | TCAGTTGCTGCCCATTCATCA | 62 |
| *mGCKfwd1* | *AGGAGGCCAGTGTAAAGATGT* | 60.5 | mEcadfwd1 | CAGTTCCGAGGTCTACACCTT | 60.9 |
| *mGCKrev1* | *CTCCCAGGTCTAAGGAGAGAAA* | 60 | mEcadrev1 | TGAATCGGGAGTCTTCCGAAAA | 61.4 |
| mGCKfwd4 | TGAGCCGGATGCAGAAGGA | 63 | mEcadfwd4 | CAGGTCTCCTCATGGCTTTGC | 63 |
| mGCKrev4 | GCAACATCTTTACACTGGCCT | 60.6 | mEcadrev4 | CTTCCGAAAAGAAGGCTGTCC | 60.9 |
| mNeurod1fwd3 | ACAGACGCTCTGCAAAGGTTT | 62.5 | mAKT1fwd1 | ATGAACGACGTAGCCATTGTG | 60.7 |
| mNeurod1rev3 | GGACTGGTAGGAGTAGGGATG | 60.2 | mAKT1rev1 | TTGTAGCCAATAAAGGTGCCAT | 60 |
| mNeurod1fwd4 | ATGACCAAATCATACAGCGAGAG | 60.2 | mAKT1fwd3 | AGAAGAGACGATGGACTTCCG | 61 |
| mNeurod1rev4 | TCTGCCTCGTGTTCCTCGT | 62.9 | mAKT1rev3 | TCAAACTCGTTCATGGTCACAC | 60.7 |
| *mNkx6.1fwd3* | *CAAGGGGACTTCGGAGAATGA* | 61.2 | mGSK3bfwd1 | TGGCAGCAAGGTAACCACAG | 62.4 |
| *mNkx6.1rev3* | *ACCGCTCGATTTGTGCTTTTT* | 61.4 | mGSKbrev1 | CGGTTCTTAAATCGCTTGTCCTG | 61.7 |
| mNkx6.1fwd4 | CTGCACAGTATGGCCGAGATG | 62.9 | mCyclophilin A fwd1 | GAGCTGTTTGCAGACAAAGTTC | 60.2 |
| mNkx6.1rev4 | CCGGGTTATGTGAGCCCAA | 61.7 | mCyclophilin A rev1 | CCCTGGCACATGAATCCTGG | 62.6 |
| *mFoxa2fwd3* | *GGAGGCAAGAAGACCGCTC* | 62.4 |  |  |  |
| *mFoxa2rev3* | *CCTTTAGCTCGCTTAGGCCAC* | 62.8 |  |  |  |
| mFoxa2fwd4 | CCCTACGCCAACATGAACTCG | 63 |  |  |  |
| mFoxa2rev4 | GTTCTGCCGGTAGAAAGGGA | 61.2 |  |  |  |
| mHnf1afwd1 | GACCTGACCGAGTTGCCTAAT | 61.5 |  |  |  |
| mHnf1arev1 | GCGAAGTCTTCCCCATCGTC | 62.6 |  |  |  |
| mHnf1afwd2 | GTGGCGAAGATGGTCAAGTC | 60.7 |  |  |  |
| mHnf1arev2 | GCGTGGGTGAATTGCTGAG | 61.4 |  |  |  |
| mHnf4afwd2 | AAGGTGCCAACCTCAATTCATC | 60.8 |  |  |  |
| mHnf4arev2 | CACATTGTCGGCTAAACCTGC | 61.8 |  |  |  |
| mHnf4afwd4 | CACGCGGAGGTCAAGCTAC | 62.7 |  |  |  |
| mHnf4arev4 | CCCAGAGATGGGAGAGGTGAT | 62.2 |  |  |  |

Table 1 – RT-PCR primers utilised in the study. The table shows the primer name, sequence and TM for each primer set used. Cyclophilin A and HPRT1 were utilised as housekeeping genes.
